# Spatially and Temporally Resolved Measurements of NO Adsorption/Desorption over NOx‐Storage Catalyst

**DOI:** 10.1002/cphc.202000765

**Published:** 2020-11-13

**Authors:** Sui Wan, Yiran Guo, Thomas Häber, Rainer Suntz, Olaf Deutschmann

**Affiliations:** ^1^ Karlsruhe Institute of Technology Institute of Catalysis Research and Technology (IKFT) Hermann-von-Helmholtz-Platz 1 76344 Eggenstein-Leopoldshafen Germany; ^2^ Karlsruhe Institute of Technology Institute for Chemical Technology and Polymer Chemistry (ITCP) Engesserstr. 20 76128 Karlsruhe Germany

**Keywords:** mass-transfer limitation, NOx storage catalyst (NSC), phase-correlated recording, planar laser-induced fluorescence (PLIF), spatiotemporal distribution

## Abstract

The two‐dimensional (2D) temporal evolution of the NO‐concentration over a NOx‐storage catalyst is investigated in situ with planar laser‐induced fluorescence (PLIF) in an optically accessible parallel wall channel reactor. Signal accumulated phase‐correlated 2D‐recordings of repetitive adsorption/desorption cycles are obtained by synchronizing the switching of the NO gas flow (on/off) with the laser and detection system, thereby significantly increasing the signal‐to‐noise ratio. The gas compositions at the reactor outlet are additionally monitored by ex‐situ analytics. The impacts of varying feed concentration, temperature and flow velocities are investigated in an unsteady state. Transient kinetics and the mass transfer limitations can be interpreted in terms of the NO concentration gradient changes. The technique presented here is a very useful tool to investigate the interaction between surface kinetics and the surrounding gas flow, especially for transient catalytic processes.

## Graphical abstract

**The spatial and temporal evolution** of the NO distribution over an NOx‐storage catalyst is obtained via phase‐correlated planar laser‐induced fluorescence. It synchronizes flow‐controlling valves and a laser system with a programmable timing unit (PTU). The presented technique is an attractive tool to understand the interaction between kinetics and mass transfer, especially for unsteady catalytic processes.

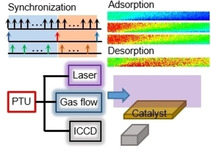

Besides selective catalytic reduction (SCR), the NOx‐storage or the combination of both is considered as one of the most promising concepts for vehicles to meet the criteria of stricter NOx emission limits under lean‐combustion conditions. NOx storage catalysts (NSCs) are based on the concept of storing NOx in the catalyst as nitrates or nitrites via alkaline‐earth metals under fuel‐lean conditions.^[1]^ The catalyst must periodically be regenerated by switching to fuel‐rich conditions, thereby releasing the stored NOx. In such a dynamic system, the catalyst changes its activity in space and time due to spatial and temporal variations of the fluid phase and the time of operation. Numerical simulations today provide temporally resolved 2D or 3D gas‐phase species profiles over NSCs,^[2]^ however, no corresponding spatially and temporally resolved experimental data has so far been provided for model validation.

A common approach for analyzing the gas phase in catalytic reactors is the gas sampling technique. In recent years, capillary sampling techniques have been used to obtain spatially resolved 1D‐concentration profiles of gas‐phase species in the flow direction.^[3]^ Although flow disturbances from the immersed probe tip can be decreased by using a very thin capillary, the disruptive influence cannot be completely avoided and temporal variations of the flow‐field can hardly be detected.^[4]^ Moreover, this technique provides at best one‐dimensional experimental data.

Non‐invasive laser diagnostics, such as planar laser‐induced fluorescence (PLIF), Raman spectroscopy and cavity ring‐down spectroscopy (CRDS), are widely used in combustion research to obtain spatio‐temporal gas‐phase species distributions.^[5]^ However, only very few studies have applied (planar) laser diagnostics to investigate catalytic processes. Mantzaras and co‐workers conducted several pioneering studies using PLIF and Raman to investigate catalytically supported combustion.^[6]^ Zellner et al. and Wan et al. successfully employed PLIF to visualize the 2D concentration maps of NO during the reduction by molecular hydrogen and HCHO during the catalytic oxidation over Pt/Al_2_O_3_ in a parallel wall channel, respectively.^[7]^ The investigations above were carried out under steady‐state conditions. Kang et al. visualized the HCHO distribution above a platinum catalyst under transient conditions with single‐shot PLIF.^[8]^ Zetterberg, Lundgren and co‐workers measured the temporal evolution of the 2D distributions of CO and CO_2_ over palladium.^[9]^ However, since the concentrations are usually rather low, the single‐shot PLIF images have a limited signal‐to‐noise ratio and several consecutive laser shots are averaged, resulting in an effective time resolution of about one second or more. Such a long acquisition time may not be suitable for the investigation of rapidly changing catalytic processes with high reactivity or short residence time, since the species distribution can vary significantly from laser shot to laser shot. In this work, we demonstrate spatially and temporally resolved PLIF measurements with a good signal‐to‐noise ratio and short acquisition time (temporal resolution) to investigate the transient NOx storage process.

In this study, a 25 mm long and 18 mm wide catalyst plate cut from a commercial NSC (Umicore AG & Co. KG), which contains Pt, Pd and Rd as active components as well as Ba and Ce‐ZrOx as storage components, is placed in the middle of a 2 mm high parallel wall channel (details of the experimental set‐up are given in the supporting information). Such height ensures that the hydraulic diameter of this channel is similar to that of common monolithic catalysts in the chemical industry (0.5–8 mm),^[10]^ therefore the interaction between the chemical kinetics and diffusion revealed in this study is comparable to the true catalyst. Reducing the system to a 2D‐problem will allow us to understand and validate detailed numerical models against spatial and time‐resolved concentration profiles and to transfer those models to the actual 3D honeycomb structures.

The investigation of the NO adsorption/desorption process at ambient pressure starts with flowing a gas mixture without NO consisting of 1 % O_2_, balanced with N_2_, over the fully reduced catalyst for 10.4 s. NO of different concentration is then added for 62.5 s and afterwards the inlet feed is changed back to the NO free 1 % O_2_ in N_2_ mixture. Flow switching is realized using two synchronized 3/2‐way solenoid valves (Buerkert, Type0330). The residence time of the gas flow over the catalyst in this study is less than 50 ms.

For the PLIF measurements, NO is excited by a thin laser sheet at a wavelength of 226.668 nm,^[7a]^ and its fluorescence is captured by an ICCD camera (LaVision). Preliminary experiments confirmed that the NO‐fluorescence signal intensity is in the linear regime and consequently proportional to the laser energy and to the molar concentration of NO (see supplementary material). Leaving density effects aside, no temperature dependence of the fluorescence is found. Since the O_2_‐concentration is approximately two orders of magnitude higher than that of the reactant NO and because the product NO_2_ concentration is very low (<20 ppm), self‐quenching of NO and quenching by NO_2_ are negligible. Because of the low nitrogen oxide mole fractions, the O_2_ concentration does not vary in the entire process, thus, the O_2_ quenching is a constant factor and independent of the spatial location in the channel. Therefore, absolute NO concentrations are accessible by taking reference images under identical conditions (inlet feed composition, temperature, …) leaving out the catalytic plate in the channel:

(1) $$C\left(x,y\right)=C_{ref}\frac{I(x,y)}{I_{ref}\left(x,y\right)}\frac{P_{ref}}{P}.$$

Here I(x,y) is the fluorescence signal intensity for each pixel and P is the laser pulse energy. The subscript ref stands for the reference image and $C_{ref}$ , the inlet feed concentration, is derived from the mass‐flow controller settings and is additionally monitored by independent FTIR measurements. The reference image $I_{ref}\left(x,y\right)$ also takes spatial inhomogeneities of the laser profile as well as in the sensitivity of the ICCD camera into account. The outlet gas flow is additionally analyzed by FTIR and an O_2_ sensor to verify the PLIF results and to monitor the concentrations of other species at the outlet.

Due to the low NO concentration in combination with the strong quenching by O_2_, the fluorescence intensity and, hence, the signal‐to‐noise ratio (SNR) is quite low under single laser shot conditions. For a flow with 150 ppm NO the SNR is about 7. Here, the SNR is defined as the mean NO‐PLIF‐signal in an inert channel with a uniform NO distribution divided by its standard deviation in space. Thus, it is the upper limit for the SNR in a single image. One can increase the SNR by averaging multiple images by $\sqrt{N}$ , either by taking consecutive images N in a time series or by repeating the cycle several times and accumulate the signal phase‐locked at different phase steps, respectively. Depending on the timescale of the transient process under investigation, the first approach is not feasible or limited to a small number of images without losing too much information or even falsify the temporal evolution of the processes under investigation in the time domain. The second approach has no such limitations, but requires that the process is reproducible and that the images are captured at the same phase within each cycle. Such phase‐correlated recordings are obtained in this study by synchronizing the flow switching valves (NOx on/off) with the laser pulses and the gating of the ICCD camera (details are given in the supporting information).

The results presented in this study are recorded at a repetition rate of 9.6 Hz according to time steps of 208 ms at a gating time of 200 ns. The latter is chosen according to the laser‐pulse duration and the fluorescence lifetime. Intermediate time steps for signal acquisition are easily accessible by introducing a time delay, if necessary. Thus, the time resolution is only limited by the fluorescence lifetime and the temporal characteristics of the flow switching setup. Here we demonstrate the advantages of phase‐locked imaging of transient catalytic processes, especially at the onset of the storage cycle, as well as the combination of phase‐locked and temporal averaging to greatly improve the signal‐to‐noise ratio.

Figure 1 shows the inlet and outlet concentrations as a function of time at T=723K and a mass flow rate of ${\dot{V}}_{in}=1\;\;slm$ . The valves switch the gas mixture at t=0s , adding 390 ppm NO to the inlet feed, and switch back to the mixture without NO at *t*=62.5 s. The switching timings are indicated by vertical dashed lines. Figure 1 contains data from both, the PLIF measurements and the ex‐situ FTIR analysis. The PLIF data are the averaged NO concentrations upstream (inlet) and downstream (outlet) of the catalytic plate perpendicular to the flow direction. Overall, NO concentrations at the inlet and the outlet measured by PLIF and FTIR are in good agreement with each other and differ only in the rise and fall time when the NO feed is turned on or off. The latter is due to a larger dead volume in the FTIR cell and streamwise diffusion in the connection pipe. The propagation delay of the gas sample caused by the 2 m long pipe between the catalytic cell and the FTIR has already been taken into account. As an in‐situ method PLIF instantaneously responds to concentration changes in the channel. According to the PLIF‐results, first NO‐signal is observed at t=1.67s in front of the catalyst plate, and it increases fast to 390 ppm as reaching the steady‐state within 10 seconds. The delay and the concentration fluctuation at t=∼5s are caused by the unavoidable dead volume between the controlling valves and the catalytic channel as well as by diffusion processes. Such fast NO concentration changes cannot be monitored by averaging consecutive PLIF images.

**Figure 1:**
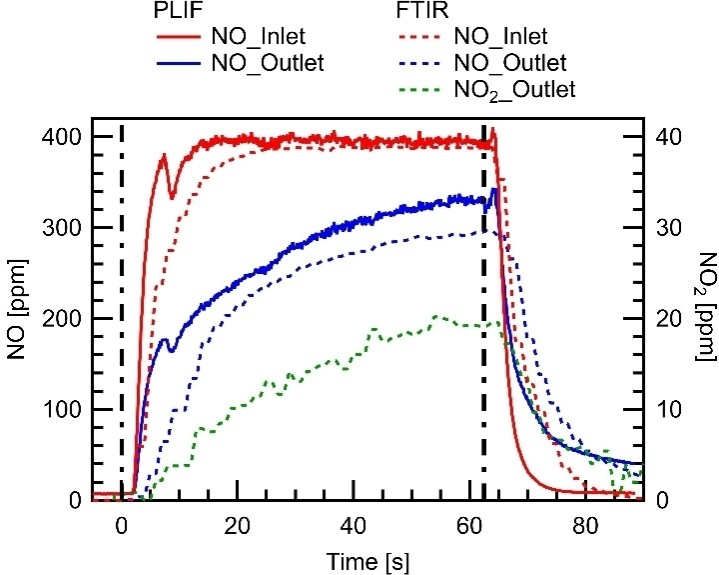
Inlet and outlet NO concentration measured at T=723K , ${\dot{V}}_{in}=1\;\;slm$ measured with PLIF (in situ) and FTIR (ex situ). In addition, the NO_2_ concentration at the outlet, measured via FTIR, is provided. At t=0s the feed gas is switched from the O_2_/N_2_ gas mixture to the NO/O_2_/N_2_ gas mixture. At t=62.5s the feeding gas is switched back to the O_2_/N_2_ gas mixture.

As shown in Figure 1, at smaller detection times (t<30 s), NO storage is very effective, indicated by a large NO concentration difference between the inlet and the outlet. In the further course of the NO storage period, the NO concentration difference between the inlet and the outlet decreases over time but remain far from reaching steady‐state conditions (outlet concentration=inlet concentration) within the time scale of the experiment.

Additionally, the FTIR data show an increasing concentration of desorbed NO_2_ up to 20 ppm, but no other nitrogen oxides are detected. The results indicate that the NOx storage efficiency decreases over time due to the reduced number of available active sites. After the NO supply has been turned off (t=62.5s ), some desorbed NO and NO_2_ are detected at the outlet (concentration in outlet greater than for the inlet). The amount decreases over time, but ∼40 ppm NO is still detected at the exit at t=90s .

The spatio‐temporal evolution is visualized using 2D NO‐concentration maps obtained by PLIF, as shown in Figure 2. The catalyst plate is located at the bottom of the channel between x=0mm and 25mm . All images represent the average over 10 storage cycles at the indicated time. NO concentration gradients are observed in both, streamwise and wall‐normal direction, due to the interaction of catalytic surface reactions with the NO diffusion and convection in wall‐normal and streamwise direction, respectively. During the NO storage phase (0∼62.5 s), lower NO concentrations are found just above the catalytic plate than at the top of the channel, showing the diffusion is towards the catalyst. After the NO supply is stopped (62.5 s), the NO concentration at the catalytic surface is higher, which indicates that desorbed NO diffuses away from the catalytic plate.

**Figure 2:**
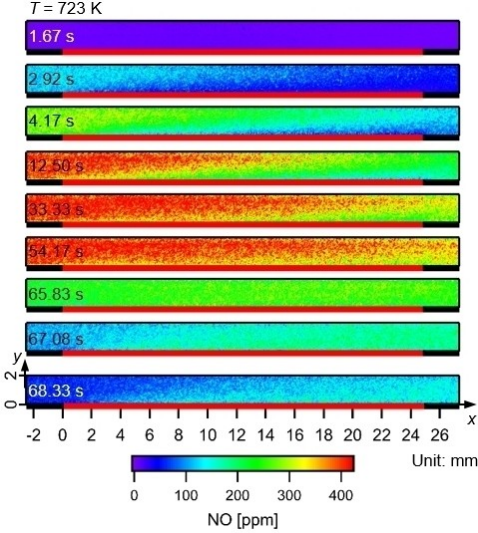
Spatio‐temporal development of the NO distribution over a NOx storage catalyst at T=723K and ${\dot{V}}_{in}=1\;\;slm$ . The position of the 25 mm long catalyst plate is marked as the red solid line at the bottom of each image.

At the catalytic surface, without considering the Stefan flow due to its much smaller value compared to diffusion, the net NO adsorption rate is equal to the NO mass flux driven by diffusion. Moreover, since the concentration gradient in streamwise direction is much smaller compared to the gradient in wall‐normal direction, we can simplify the species transport equation for NO as follows:

(2) $$k_{ads}N_{s}C_{NO}=D{\left.\frac{dC_{NO}}{dy}\right|}_{y=0},$$

where $k_{ads}$ is the rate coefficient of the net adsorption reaction, $N_{s}$ is the number of the empty sites, $C_{NO}$ is the local NO concentration and D is the diffusion coefficient. Since D is constant at a given temperature, the wall‐normal NO concentration gradient is a measure of the net NO adsorption rate. Measured wall‐normal NO‐concentration profiles at x=5mm at different times during the storage phase are plotted in Figure 3. Early in the storage phase, where the inlet concentration is still increasing (t<10s ), the NO concentration at the top of the channel rises faster than at the catalytic plate. Therefore, the NO concentration gradient, which is the driving force for diffusion, increases over time, indicating that the reaction rate is limited by diffusion in this period. The corresponding profiles are phase‐locked averaged over ten storage cycles. A temporal averaging over consecutive images is not possible in this early and dynamic storage period, because spatial variations of the NO distribution are quite fast, compare e. g. the open marks.

**Figure 3:**
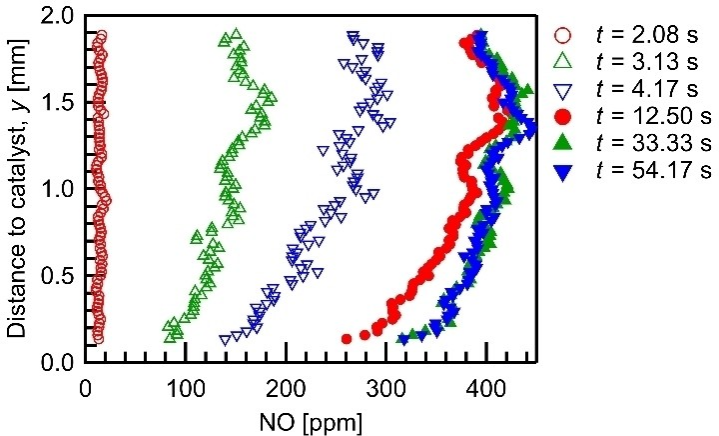
NO concentration profiles in wall‐normal direction at x=5mm (T=723K , ${\dot{V}}_{in}=1\;\;slm$ ).

At later times (t>10s ) the temporal concentration change at the reactor outlet is only ≈3ppm/s (compare Figure 1). Therefore, to further improve the SNR, a combination of phase‐locked and temporal averaging is employed. The total number of the images used for averaging is enlarged to 110, by taking 11 consecutive laser shots (Δt=1.04s ) of each cycle for ten storage cycles, respectively. The results show the NO concertation gradients decrease over time, indicating that the surface reaction rate slows down due to the reduced number of the empty sites.

Since the adsorption rate coefficient and the diffusion coefficient are constant at a given temperature, from Eq. 2, one obtains

(3) $$N_{s}∼\frac{1}{C_{NO}}{\left.\frac{dC_{NO}}{dy}\right|}_{y=0}$$

in the vicinity of the catalyst. PLIF measured wall‐normal NO concentration profiles as shown in Figure 3 are fitted to parabolic curves, which has been proved to be sufficient to describe the gas flow over the catalyst.^[7a]^ At each time‐step, single shots from 10 storage cycles are used for phase‐locked averaging. The values of $\left(dC_{NO}/dy\right)/C_{NO}$ calculated from the fitting curves over time at x=5mm and 20mm and y=0mm are plotted in Figure 4. The results show that, after feeding 390 ppm NO for 62.5 s at T=723K , the number of empty sites is reduced by over 70 %. Despite the noise level, a tendency of the faster reduced number of empty sites can be observed within the first 10 seconds, when the NO inlet concentration is still rapidly increasing. A higher value of $\left(dC_{NO}/dy\right)/C_{NO}$ is found at x=20mm than that at x=5mm in the first 35 s, indicating the surface coverage is higher upstream due to the higher NO concentration. As the overall reaction rate starts to be limited by the number of empty sites, the NO concentration becomes relatively uniform just above the catalytic plate as shown in Figure 2 at t=54.17s , the value of $\left(dC_{NO}/dy\right)/C_{NO}$ becomes similar at x=5mm and 20mm .

**Figure 4:**
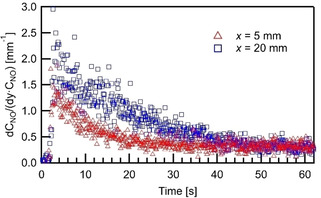
$/C_{NO}$ calculated from the parabolic fitting curve of the PLIF data in the vicinity of catalyst (T=723K , ${\dot{V}}_{in}=1\;\;slm$ ).

The impacts of flow rate, operating temperature and feed concentration on the NO‐distribution are presented in Figure 5. As shown in Figure 5(a) and (b), with a smaller flow rate, the decrease of the concentration in streamwise direction is much faster, because of the longer residence time of NO over the catalyst plate. Comparing Figure 5(b) and (d), it is found that NO concentration gradients in both, streamwise and wall‐normal direction, increase with increasing temperature. Due to thermal expansion, the flow velocity increases at higher temperatures, resulting in a decreased residence time. Nevertheless, the overall NO consumption is still higher at higher temperatures, which could be caused by both, higher reactivity and enhanced diffusion. Both effects over‐compensate the reduced residence time. After turning off the NO feed, the desorption is fast at high temperatures, as shown in Figure 5(c) and (e). Finally, in Figure 5(d) and (f) NO distributions at different NO feed concentrations are compared, while keeping the O_2_ concentration constant at 1 % (${\dot{V}}_{in}=1\;slm$ , T=698K , t=33s ). At a feed concentration of 388 ppm, the concentration decreases by 182 ppm between the inlet and outlet, so 47 % of the NO is stored. However, with a feed concentration of 155 ppm, the NO concentration is decreased by 91 ppm, corresponding to a storage of 59 %. The percentage of stored NO (conversion) is higher at the lower feed concentration, but the overall storage rate (NO stored per time) is much faster at the higher feed concentration. This indicates that the overall reaction rate is limited by the diffusion in the gas phase. The concentration gradient in wall‐normal direction increases with increasing feed concentration, resulting in a larger mass flux towards the catalytic surface.

**Figure 5:**
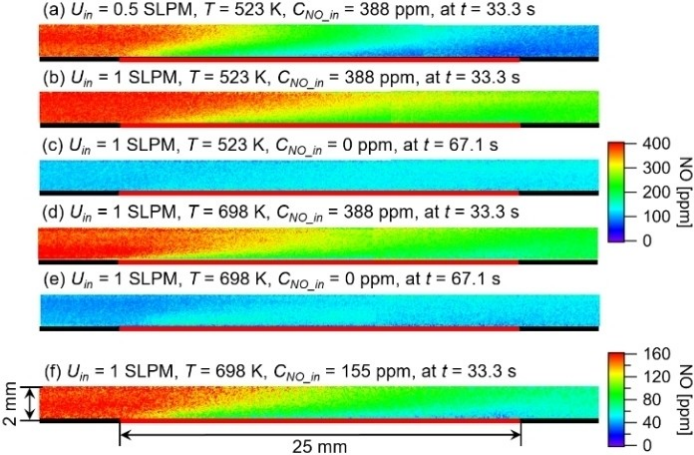
Influence of flow rate, temperature and feed concentration on the NO distribution over a NOx‐storage catalyst. (a)–(e) colour scale: 0-400ppm ; (f) color scale: 0-160ppm .

Planar laser‐induced fluorescence imaging with synchronized flow control is demonstrated in this study as a viable tool to study catalytic systems, especially under transient conditions. The spatial and temporal development of the absolute NO concentration distributions are obtained over a NOx storage catalyst, using a phase‐correlated recording method, which significantly improves the SNR by accumulating the signal over multiple adsorption/desorption cycles. The reduced catalyst reactivity over time caused by the decreased number of available sites is visualized. The overall reaction rate is under combined effects of surface kinetics as well as advective and diffusive mass transfer, but in most cases limited by gas phase diffusion.

## Conflict of interest

The authors declare no conflict of interest.

## Supporting information

As a service to our authors and readers, this journal provides supporting information supplied by the authors. Such materials are peer reviewed and may be re‐organized for online delivery, but are not copy‐edited or typeset. Technical support issues arising from supporting information (other than missing files) should be addressed to the authors.

Supplementary
