# Supplementary material for: Spatially and Temporally Resolved Measurements of NO Adsorption/Desorption over NOx‐Storage Catalyst
Source: Chemphyschem. 2020 Nov 13;21(23):2497–501. doi: 10.1002/cphc.202000765 (PMC7756476; doi:10.1002/cphc.202000765)
Supplement: Supplementary file 1 — Supplementary [file CPHC-21-2497-s001.pdf]

ChemPhysChem

Supporting Information

Spatially and Temporally Resolved Measurements of NO Adsorption/Desorption over NOx-Storage Catalyst

Sui Wan, Yiran Guo, Thomas Häber, Rainer Suntz,* and Olaf Deutschmann*

Supporting Information

Abstract: Experimental setup is described with more details. And a movie of the measurements is provided.

Table of Contents

Experimental Section, Page 2

Experimental Section

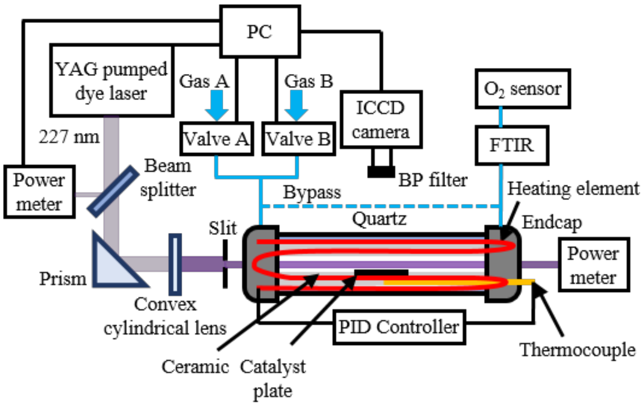

Figure S1. Schematic of the experimental setup for NO-PLIF measurements.

A schematic of the experimental setup is shown in Figure S1. It uses the same reactor as in our previous studies.[1] The inner channel has a height of 2 mm, a depth of 18 mm and a length of 150 mm. The catalyst plate (18 mm × 25 mm), cut from a commercial NSC (Umicore AG & Co. KG), is placed in an indentation located in the middle of the parallel wall channel. The reactor is optically accessible, and the temperature is controllable between room temperature and 723 K. The gas exiting reactor at its outlet is analyzed by a Fourier-transform infrared (FTIR) spectrometer and an O_2 sensor.

An injection seeded Nd:YAG laser (Quanta-Ray Pro, Spectra-Physics) is operating simultaneously at the second and third harmonics (550 mJ/pulse at 532 nm, 530 mJ/pulse at 355 nm). The second harmonic pumps the Dye laser (PrecisionScan, Sirah), which runs on DCM dye and ethanol as solvent. The laser radiation from DCM at ~ 627 nm is sum frequency mixed with the third harmonic of the YAG laser to produce a UV beam at ~ 227 nm with a pulse energy of up to 12 mJ. The beam has been split into two beams by a window. One of the beams is steered by UV-fused silica prisms, shaped into a ~ 0.3 mm thin laser sheet with a cylindrical lens (f = 500 mm) and sent through the reactor, with the laser sheet oriented perpendicular to the catalyst plate. The other beam (~ 4% total laser power) is sent to a power meter for monitoring the pulse energy fluctuation. The laser is tuned to 226.68 nm (P2(12.5) + Q12(12.5) line) to obtain the NO $A \rightarrow X$ emission.[1a] The laser-induced fluorescence is captured by an image-intensified charge-coupled device (ICCD) camera (IRO 25 & Imager QE, LaVision). An UV objective ($f = 100$ mm, $f/2.8$, LaVision) with a 75 mm extension tube and a band-pass filter (BP) at 248 nm ($\mathrm{FWHM} = 10$ nm) is attached to the ICCD camera.

An embedded programmable timing unit (PTU X, LaVision) is used to generate the pulses for synchronizing the hardware of the PLIF system (laser, camera, power monitor, etc.), and the flow as well. More details about the synchronization scheme are shown in Figure S2. The switching of the gas feed is realized via two 3/2-way solenoid valves (Buerkert), which are installed in front of the reactor. The valve switching time is $8 \sim 12$ ms. Besides the trigger signals to laser and imaging systems, the PTU inside the system computer provides an additional trigger to a programmable logic controller (SIMATIC, Siemens) with relay, which counts the incoming pulses and outputs two 5 ms triggers for controlling the valves at the prescribed timing. The timing sequence used in this study for the investigation of NO adsorption/desorption is shown in Fig. S2 (b). The laser and the camera are operating at a frequency of 9.6 Hz.

Before each recording, the catalyst is first reduced by flowing 1% H_2 in N_2 mixture for 15 min and subsequently another 5 min Gas A, 1% O_2 balanced by N_2 mixture. In the meanwhile, Gas B, a gas mixture of NO, O_2 and N_2, flows to the exhaust. This state continues until the PLC received the 100th input pulse from the PTU. The PLC generates the Trigger 1 at the 100th input pulse, which switches the Gas A flowing to the exhaust and Gas B flowing to the reactor. After counting another 600 incoming pulses, the PLC generates Trigger 2, to switch the flow back to the initial setting. A delay can be set between the trigger to the laser system and to the flow control. Therefore, it is possible to image any desired time step of the process by setting a corresponding delay. The temporal resolution is about ten milliseconds, and currently limited by the valve switching time. Since no delay is set between the triggers to the laser and PLC in this study, the 50th image is taken at the same moment as the valves acting.

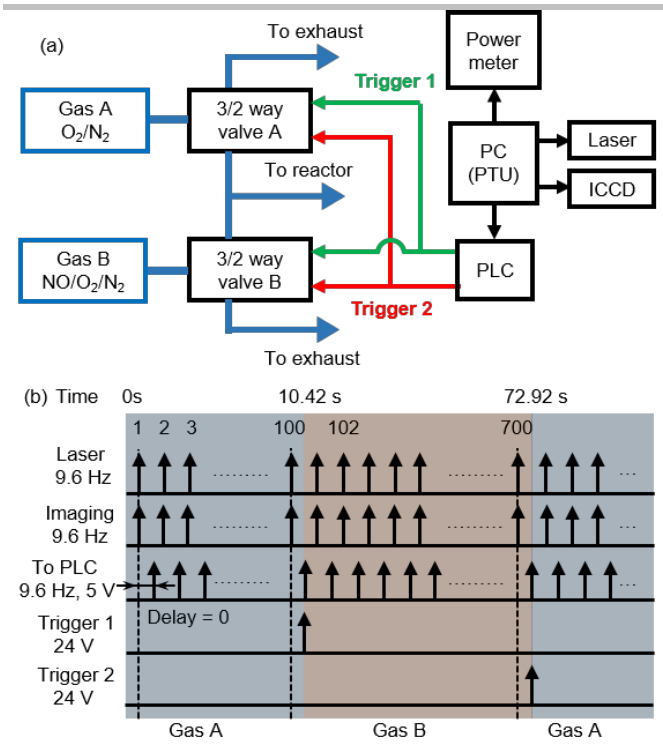

Figure S2. Synchronization scheme for transient measurements.

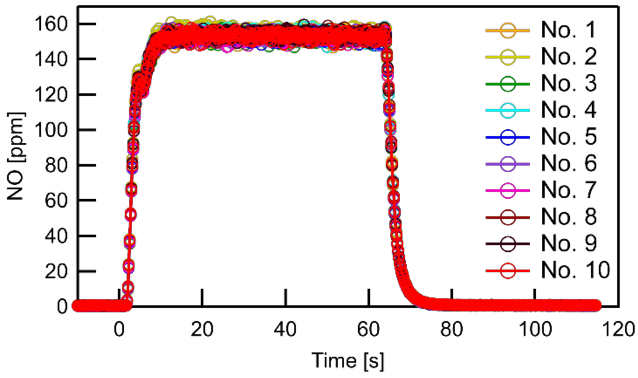

Figure S3. NO inlet concentration measured by single shot PLIF for 10 repeated cycles.

Figure S3 demonstrates the repeatability of the flow-synchronized NO PLIF measurements. The NO inlet concentration at each time point is calculated from the PLIF single shot image, by averaging the data point in the area before the catalyst plate. A good repeatability is shown from the results of 10 independent cycles, especially at the rising and falling edges where the signal intensity greatly changes between the consecutive images.

The NO fluorescence dependence on laser intensity, NO concentration and temperature are examined for the quantitative NO-PLIF measurement, and so is the quenching effect. The dependence of fluorescence signal on laser intensity is shown in Figure S4. The measurement is conducted at 473 K in an inert channel with 388 ppm NO balanced by N_2. The signal intensity is an averaged value over the pixels in the laser sheet illuminated region. The image for extracting the signal intensity is averaged over 100 shots, and a background image has also been subtracted. Very good linearity is found between the HCHO fluorescence and the laser intensity, and no saturation is observed in the investigated range of laser energies. All measurements in this paper are conducted with a laser pulse energy less than 9 mJ/pulse.

Figure S5 shows the relation between the fluorescence signal and NO concentration. The measurement is conducted at 473 K. Again, the signal intensity is the average value of the full uniform PLIF image and additionally averaged over 100 laser pulses. The laser sheet pulse energy in this measurement is 8.5 mJ/pulse. No O_2 is added for this measurement. The concentration of NO varies in the range of $50{\sim}400$ ppm. The result shows that the fluorescence signal linearly increases with an increasing NO concentration.

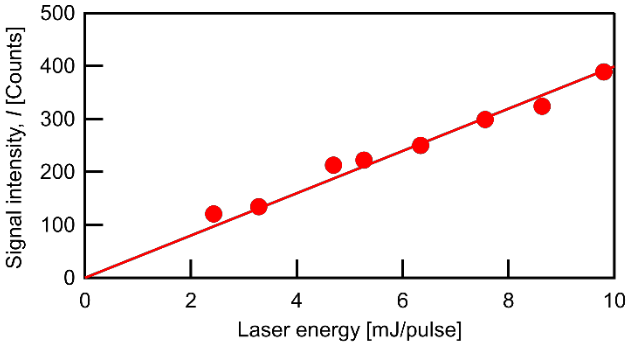

Figure S4. NO fluorescence signal intensity vs laser pulse energy.

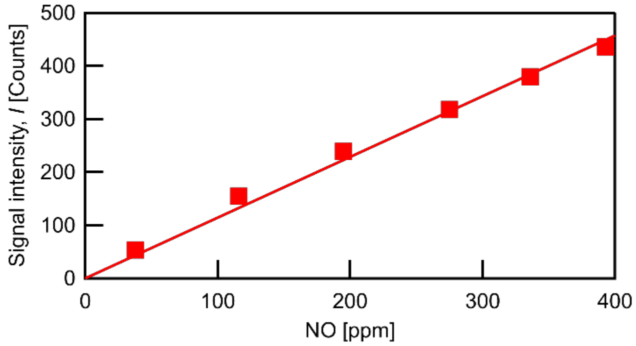

Figure S5. NO fluorescence signal intensity vs NO concentration.

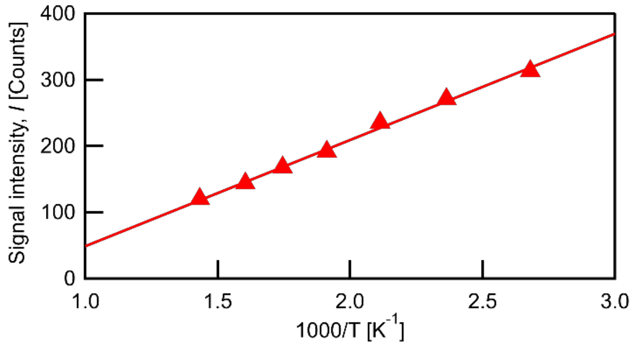

Figure S6. NO fluorescence signal intensity vs temperature.

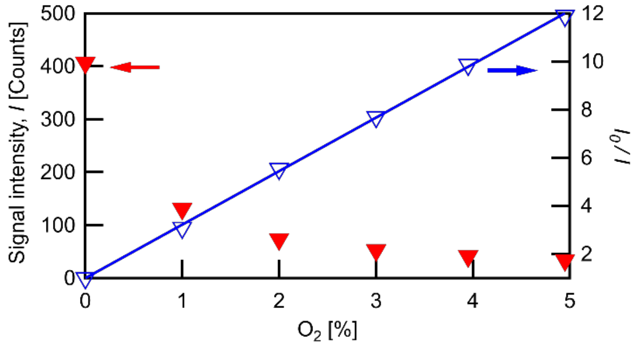

Figure S7. NO fluorescence decrease in the presence of O_2 and the Stern-Volmer plot.

A good linearity is also shown between NO fluorescence signal intensity and the reciprocal for temperature in Figure S6, when a constant NO mole fraction is used. According to the ideal gas law, the results indicate that no temperature dependence is found in the examined temperature range (423 ~ 698 K).

O_2 has long been recognized to have a strong quenching effect on fluorescence. Figure S7 shows the relationship between the NO fluorescence signal and the O_2 concentration. Measurements are conducted with a fixed NO concentration (388 ppm), while the O_2 concentration increased from 0 to 5 %. It shows that the NO fluorescence signal largely decreases with an increased O_2 concentration. The fluorescence is quenched by ~ 68% in a gas mixture containing 1% O_2. The average collisional quenching rate Q is expressed as

$$Q = \sum \sigma_{Qi} \langle v_i \rangle N_i \, , \quad (1)$$

where σ_{Qi} is the collisional quenching cross section of species i, $<v_i>$ is the average heat collision speed of the collision partner i, and N_i is the number density of the collision partner i. According to Ref. [2], at T = 400 ~ 700 K, the collisional quenching cross section of N_2, O_2, NO and NO_2 are around 0.1, 40, 23 and 95 $Å^2$ respectively. In this study, N_2, as a carrier gas, always has a concentration larger than 98%; O_2, as one of the reactant, has a constant mole fraction of 1%; NO as the other reactant has a maximum concentration less than 400 ppm. As a product, the NO_2 concentration detected by the FTIR is less than 8 ppm, four orders of magnitude lower than that of O_2. As a result, the collisional quenching contributed by NO and NO_2 is less than 5% of that from O_2 and N_2, which has negligible impact on the fluorescence quantum efficiency. In addition, since the maximum NO concentration in this study is less than 4% of the O_2 concentration, the O_2-concentration should vary less than 2% and consequently the O_2-quenching can be considered as a constant factor. The same applies for N_2.

References

[1] a) A. Zellner, R. Suntz, O. Deutschmann, *Angew. Chemie Int. Ed.*, **2015**, *54*, 2653–2655; b) S. Wan, B. Torkashvand, T. Häber, R. Suntz, O. Deutschmann, *Appl. Catal., B.*, **2020**, *264*, 118473.

[2] a) M. Tamura, P.A. Berg, J.E. Harrington, J. Luque, J.B. Jeffries, G.P. Smith, D.R. Crosley, *Combust. Flame.*, **1998**, *114(3-4)*, 502–514; b) P.H. Paul, J.A. Gray, J.L. Durant Jr, J.W. Thoman Jr., *AIAA Journal*, **1994**, *32(8)*, 1670–1675; c) M.C. Drake, J.W. Ratcliffe, *J. Chem. Phys.*, **1992**, *98(5)*, 3850–3865.

Author Contributions

All authors contributed equally to this work.
